# LncRNA KCNQ1OT1 promotes the development of diabetic nephropathy by regulating miR-93-5p/ROCK2 axis

**DOI:** 10.1186/s13098-021-00726-4

**Published:** 2021-10-15

**Authors:** Li Zhao, Huaqian Chen, Lin Wu, Zhengdong Li, Ren Zhang, Yan Zeng, Tao Yang, Hualing Ruan

**Affiliations:** 1 Department of Nephrology, Affiliated Dongfeng Hospital, Hubei University of Medicine, Shiyan, Hubei People’s Republic of China; 2 Department of Nephrology, Sinopharm Hanjiang Hospital, Hubei University of Medicine, Shiyan, Hubei People’s Republic of China; 3 Department of Endocrinology, Zhangwan District, Affiliated Dongfeng Hospital, Hubei University of Medicine, No. 16 Daling Road, Shiyan, Hubei China

**Keywords:** Diabetic nephropathy, KCNQ1OT1, miR-93-5p, ROCK2

## Abstract

**Background:**

Long non-coding RNAs (lncRNAs) have been reported to play vital roles in diabetic nephropathy (DN). The aim of this study was to explore the function of mechanism of lncRNA KCNQ1 opposite strand/antisense transcript 1 (KCNQ1OT1) in DN.

**Methods:**

DN cell models were established using high glucose (HG) treatment in human glomerular mesangial cells (HGMC) and human renal glomerular endothelial cells (HRGEC). The expression levels of KCNQ1OT1, microRNA-93-5p (miR-93-5p), and Rho associated coiled-coil containing protein kinase 2 (ROCK2) mRNA was detected by quantitative real-time polymerase chain reaction (qRT-PCR). Cell Counting Kit-8 (CCK-8) assay and flow cytometry were used to detect cell proliferation and apoptosis, respectively. ROCK2 and apoptosis/fibrosis-related protein levels were examined by western blot. The predicted interaction between miR-93-5p and KCNQ1OT1 or ROCK2 was verified by dual-luciferase reporter assay and RNA immunoprecipitation (RIP) assay.

**Results:**

KCNQ1OT1 was upregulated in DN patients and DN cell models. KCNQ1OT1 knockdown inhibited cell proliferation and fibrosis and induced apoptosis in DN cell models. MiR-93-5p was a direct target of KCNQ1OT1, and miR-93-5p inhibition restored the KCNQ1OT1 knockdown-mediated effects on cell proliferation, fibrosis and apoptosis in DN cell models. In addition, ROCK2 was identified as a target of miR-93-5p, and miR-93-5p overexpression suppressed cell proliferation and fibrosis and accelerated apoptosis by targeting ROCK2 in DN cell models. Moreover, KCNQ1OT1 regulated ROCK2 expression by binding to miR-93-5p.

**Conclusion:**

KCNQ1OT1 knockdown inhibited cell proliferation and fibrosis and induced apoptosis in DN by regulating miR-93-5p/ROCK2 axis, providing potential value for the treatment of DN.

**Supplementary Information:**

The online version contains supplementary material available at 10.1186/s13098-021-00726-4.

## Introduction

Diabetic nephropathy (DN), a serious complication of diabetes mellitus, has become the primary cause of end-stage renal disease (ESRD) and mortality in people with diabetes [1]. The main pathologic characteristics of DN are persistent albuminuria, extracellular matrix (ECM) accumulation, mesangial cell proliferation, renal tubal epithelial-mesenchymal transition (EMT), glomerular hypertrophy, and kidney fibrosis or failure [2–4]. Despite great efforts have been made in the prevention and treatment of DN, it remains one of the main causes of death [5, 6]. Moreover, the mechanism in DN is still poorly understood due to its complicated pathogenesis. Therefore, it is necessary to further study its precise molecular mechanisms, which might provide novel strategies for preventing or treating DN.

Long non-coding RNAs (lncRNAs) are a kind of non-coding RNA (> 200 nucleotides) without protein-coding capacity [7–9]. Increasing studies have shown that lncRNAs can participate in diverse pathological and physiological processes [10]. Moreover, some lncRNAs have been confirmed to play critical regulatory roles in DN. For instance, lncRNA LINC00968 promoted mesangial cell proliferation and fibrosis in DN [11]. LncRNA NEAT1 overexpression accelerated mesangial cell proliferation and fibrosis as well as restrained apoptosis in DN [12]. Inversely, lncRNA TUG1 could inhibit the development of DN [13]. As for lncRNA KCNQ1 opposite strand/antisense transcript 1 (KCNQ1OT1), it has been shown to participate in DN progression and its expression was upregulated in DN [14]. However, more roles and regulatory mechanism of KCNQ1OT1 in DN remain largely unknown.

In recent years, the interaction between lncRNAs and microRNA (miRNAs) has attracted widespread attention [15]. One famous hypothesis suggests that lncRNAs can serve as competing endogenous RNA (ceRNA) via competitively binding to miRNA response elements (MREs) to segregate miRNAs from their target mRNAs [16]. MiR-93-5p has been reported to play a pivotal role in many diseases, including DN [17–19]. Moreover, Rho associated coiled-coil containing protein kinase 2 (ROCK2) acts as a contributor to promote the development of DN [20]. Interestingly, online bioinformatics database shows that there are complementary binding sequence between miR-93-5p and KCNQ1OT1 or ROCK2, while the interaction between them is not reported before. We hypothesized that KCNQ1OT1 might regulate development of DN via sponging miR-93-5p and regulating ROCK2.

In our work, we measured KCNQ1OT1, miR-93-5p and ROCK2 expression in patients with DN and DN cell models. Moreover, we investigated the impact of KCNQ1OT1 on proliferation, apoptosis and fibrosis in DN cell model. Additionally, we confirmed the ceRNA network of KCNQ1OT1/miR-93-5p/ROCK2. This study aimed to explore the pathogenesis of DN and offer a theoretical basis for DN treatment.

## Materials and methods

Blood samples from 33 DN patients and 33 paired healthy subjects were collected at Affiliated Dongfeng Hospital, Hubei University of Medicine. Blood samples were then centrifugation at 3000 rpm for 10 min. Next, the supernatant was put in a clean tube and then centrifuged at 1500 rpm for 30 min. Finally, the final supernatant was kept in a refrigerator at − 80 °C until RNA extraction. This research had acquired approval from the Research Ethics Committee of Affiliated Dongfeng Hospital, Hubei University of Medicine.

## Cell culture and transfection

Human glomerular mesangial cells (HGMC) and human renal glomerular endothelial cells (HRGEC) were bought from Sciencell Research Laboratories (Carlsbad, CA, USA) and cultured in Dulbecco’s modified Eagle’s medium (DMEM; Invitrogen, Carlsbad, CA, USA) containing 10% fetal bovine serum (FBS; Invitrogen) in a moist atmosphere with 5% CO_2_ at 37 °C. HGMC and HRGEC were treated with 5.5 mM normal glucose (NG group) or 30 mM high glucose (HG group). HG treatment was performed to mimic the DN cells.

Small interfering RNA (siRNA) against KCNQ1OT1 (si-KCNQ1OT1), miR-93-5p mimic (miR-93-5p), miR-93-5p inhibitor (anti-miR-93-5p), ROCK2 overexpression plasmid (pcDNA-ROCK2), and their matched controls (si-NC, miR-NC, anti-miR-NC, and pcDNA-con) were acquired from GenePharma (Shanghai, China). According to the recommendations, these oligonucleotides (50 nM miRNA mimic/inhibitor and 20 nM siRNA) or plasmid (2 μg) were transfected into HGMC and HRGEC using Lipofectamine 3000 reagent (Invitrogen).

### Quantitative real-time polymerase chain reaction (qRT-PCR)

Total RNA was isolated by Trizol reagent (Invitrogen). After that, complementary DNA (cDNA) was generated using PrimeScript RT Reagent Kit (TaKaRa, Dalian, China) for lncRNAs or mRNAs and Mir-X™ miRNA First-Strand Synthesis Kit (TaKaRa) for miRNAs. Next, the cDNA was diluted and subjected to qRT-PCR using SYBR Green Master Mix (Takara) through a CFX Real-time PCR system (Bio-Rad, Hercules, CA, USA). The information of primers was listed: KCNQ1OT1: 5′-CCTCCCTCACTGAGCTTTGG-3′ (forward; F) and 5′-GTGCGGACCCTATACGGAAG-3′ (reverse; R); miR-93-5p: 5′-GGGCAAAGTGCTGTTCGTG-3′ (F) and 5′-CAGTGCGTGTCGTGGAGT-3′ (R); ROCK2: 5′-TGCGGTCACAACTCCAAGCCTT-3′ (F) and 5′-CGTACAGGCAATGAAAGCCATCC-3′ (R); glyceraldehyde-3-phosphate dehydrogenase (GAPDH): 5′-CATCACTGCCACCCAGAAGACTG-3′ (F) and 5′-ATGCCAGTGAGCTTCCCGTTCAG-3′ (R); U6: 5′-AATTGGAACGATACAGAGAAGATTAGC-3′ (F) and 5′-TATGGAACGCTTCACGAATTTG-3′ (R). The RNA levels were evaluated using the 2^−ΔΔCt^ method, followed by normalization to GAPDH (for KCNQ1OT1 and ROCK2) or U6 (for miR-93-5p).

### Cell proliferation assay

Cell Counting Kit-8 (CCK-8; Keygen, Nanjing, China) was used for measuring cell proliferation. Briefly, HGMC and HRGEC suspension were added into each well of 96-well plates. After transfection, CCK-8 solution (10 μL) was placed into per well using a pipette tip and incubated for 2–3 h. Lastly, the absorbance at 450 nm was detected under a microplate reader (Bio-Rad).

### Flow cytometry

Annexin V-fluorescein isothiocyanate (FITC)/propidium iodide (PI) apoptosis detection kit (Sangon Biotech, Shanghai, China) was used for detection of apoptosis. After transfection, the cells were collected, and then incubated with Annexin V-FITC and PI for 0.5 h in the dark, followed by the detection of apoptotic cells with flow cytometry (Partec AG, Arlesheim, Switzerland).

### Western blot assay

RIPA lysis buffer (Keygen) was used for extracting total proteins. After that, the protein concentration of supernatants was tested with BCA protein assay kit (Tanon, Shanghai, China), and then proteins were subjected to sodium dodecyl sulfate polyacrylamide gel electrophoresis (SDS-PAGE) and transferred onto the polyvinylidene fluoride membranes (Bio-Rad). After blockage using 5% skim milk (Beyotime, Shanghai, China), the membranes were immunoblotted by primary antibodies for 12–14 h at 4 °C. After incubation of corresponding secondary antibody, the visualization of protein blots was achieved by an enhanced chemiluminescence kit (Keygen). The antibodies including B-cell lymphoma-2 (Bcl-2; ab194583, 1:1000), BCL2-associated X protein (Bax; ab77566, 1:1000), fibronectin (FN; ab2413, 1:1000), collagen. (Col). I (ab34710, 1:2000) Col. IV (ab19808, 1:1000), E-Cadherin (ab219332, 1:500), vimentin (ab137321, 1:500), N-Cadherin (ab98952, 1:1000), ROCK2 (ab71598, 1:1000), GAPDH (ab37168, 1:2000), and HRP-conjugated IgG anti-rabbit (ab205718, 1:4000) were purchased from Abcam (Cambridge, UK).

### Bioinformatics analysis and dual-luciferase reporter assay

The binding sites of miR-93-5p and KCNQ1OT1 or ROCK2 were predicted using starBase (http://starbase.sysu.edu.cn/) or TargetScan (http://www.targetscan.org/). The fragments of KCNQ1OT1 or ROCK2 3′UTR that contained miR-93-5p binding sequence were constructed and inserted into pmirGLO luciferase reporter vector (Promega, Madison, WI, USA), namely wild-type reporter vectors (KCNQ1OT1-WT and ROCK2 3′UTR-WT). Meanwhile, mutated-type reporter vectors (KCNQ1OT1-MUT and ROCK2 3′UTR-MUT) without binding sites were generated in the same way. After that, the constructed reporter plasmid and miR-NC or miR-93-5p were co-transfected into HGMC and HRGEC for 48 h. At last, the luciferase activity was estimated through a Dual-luciferase Reporter Assay System (Promega).

### RNA immunoprecipitation (RIP) assay

RIP assay was performed using the EZMagna RIP kit (Millipore, Billerica, MA, USA). Briefly, cell lysate was incubated with RIP immunoprecipitation buffer that contained magnetic beads coupled with Argonaute2 (Ago2) antibody and immunoglobulin G (IgG; as the control) for 6–8 h at 4 °C. After that, the beads were digested with protease K at 55 °C for 0.5 h for removing the proteins. At last, the purified RNA was further used for qRT-PCR to test the enrichment levels of KCNQ1OT1, miR-93-5p and ROCK2.

### Statistical analysis

The data were displayed as the mean ± standard deviation (SD). All data from at least 3 independent experiments were analyzed by GraphPad Prism 7 (GraphPad Software, Inc., La Jolla, CA, USA). Student’s *t*-test and a one-way analysis of variance with Tukey test were applied for analyzing the difference between two or more groups, respectively. Correlation analysis between miR-93-5p and KCNQ1OT1 or ROCK2 was performed by Pearson’s correlation coefficient. *P* < 0.05 was considered to be statistical significance.

## Results

### KCNQ1OT1 was upregulated and miR-93-5p was downregulated in DN

**Fig. 1:**
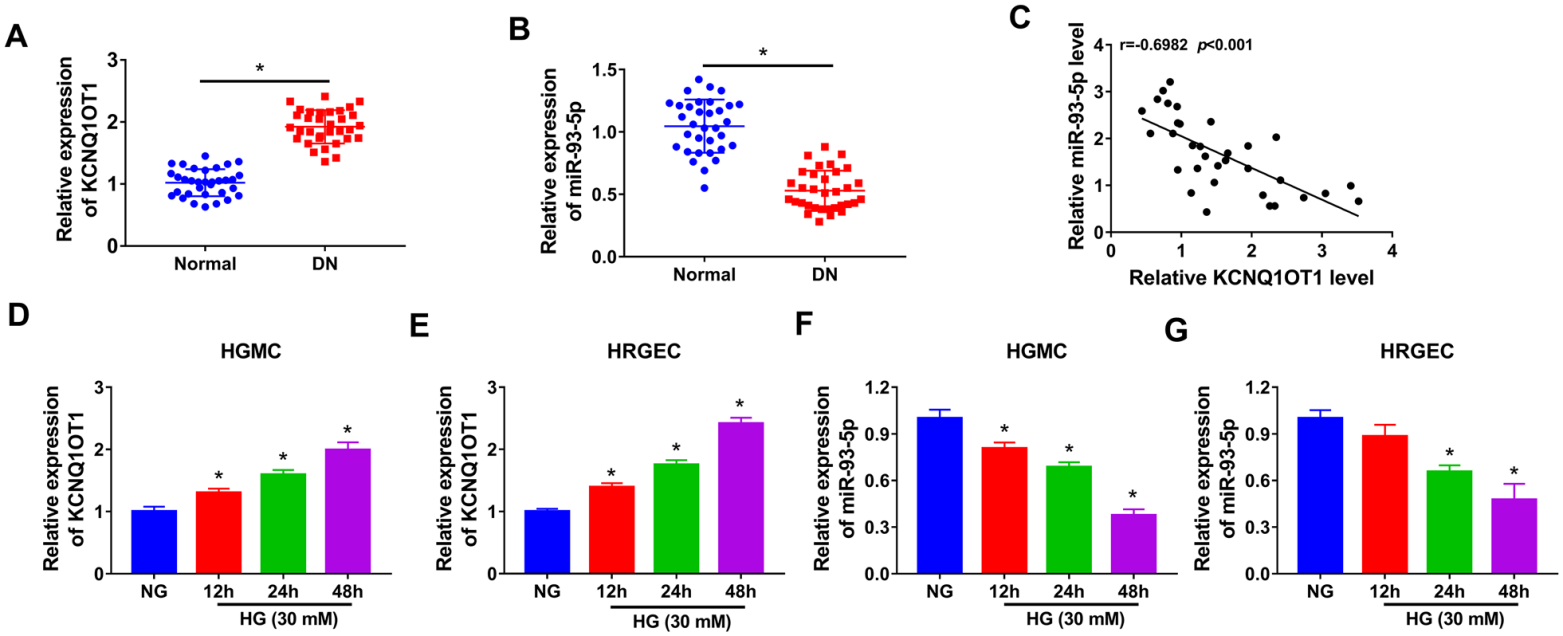
KCNQ1OT1 was overexpressed and miR-93-5p was lowly expressed in DN patients and high glucose-induced HGMC and HRGEC. **A** and **B** The expression of KCNQ1OT1 and miR-93-5p was measured by qRT-PCR in serum samples of DN patients and healthy subjects. **C** The correlation between KCNQ1OT1 and miR-93–5 expression was analyzed in serum samples of DN patients. (D-G) The levels of KCNQ1OT1 and miR-93-5p were determined by qRT-PCR HGMC and HRGEC treated with NG or HG. *P < 0.05

Firstly, KCNQ1OT1 and miR-93-5p levels in the serum of DN patients were determined by qRT-PCR. The data showed that the expression of KCNQ1OT1 was increased and the expression of miR-93-5p was decreased in the serum of DN patients compared with that in normal group (Fig. 1A and B). Moreover, we found that KCNQ1OT1 expression was negatively correlated with miR-93-5p expression in the serum of DN patients (Fig. 1C). Next, we measured the expression of KCNQ1OT1 and miR-93-5p in HGMC and HRGEC treated with NG or HG. The results showed that the expression of KCNQ1OT1 was time-dependently increased in HG-induced HGMC and HRGEC (Fig. 1D and E). In addition, miR-93-5p expression was time-dependently reduced in HG-treated HGMC and HRGEC (Fig. 1F and G). These results indicated that KCNQ1OT1 and miR-93-5p might play critical roles in DN.

### KCNQ1OT1 knockdown inhibited proliferation, ECM accumulation and EMT and induced apoptosis in HG-induced HGMC and HRGEC

**Fig. 2:**
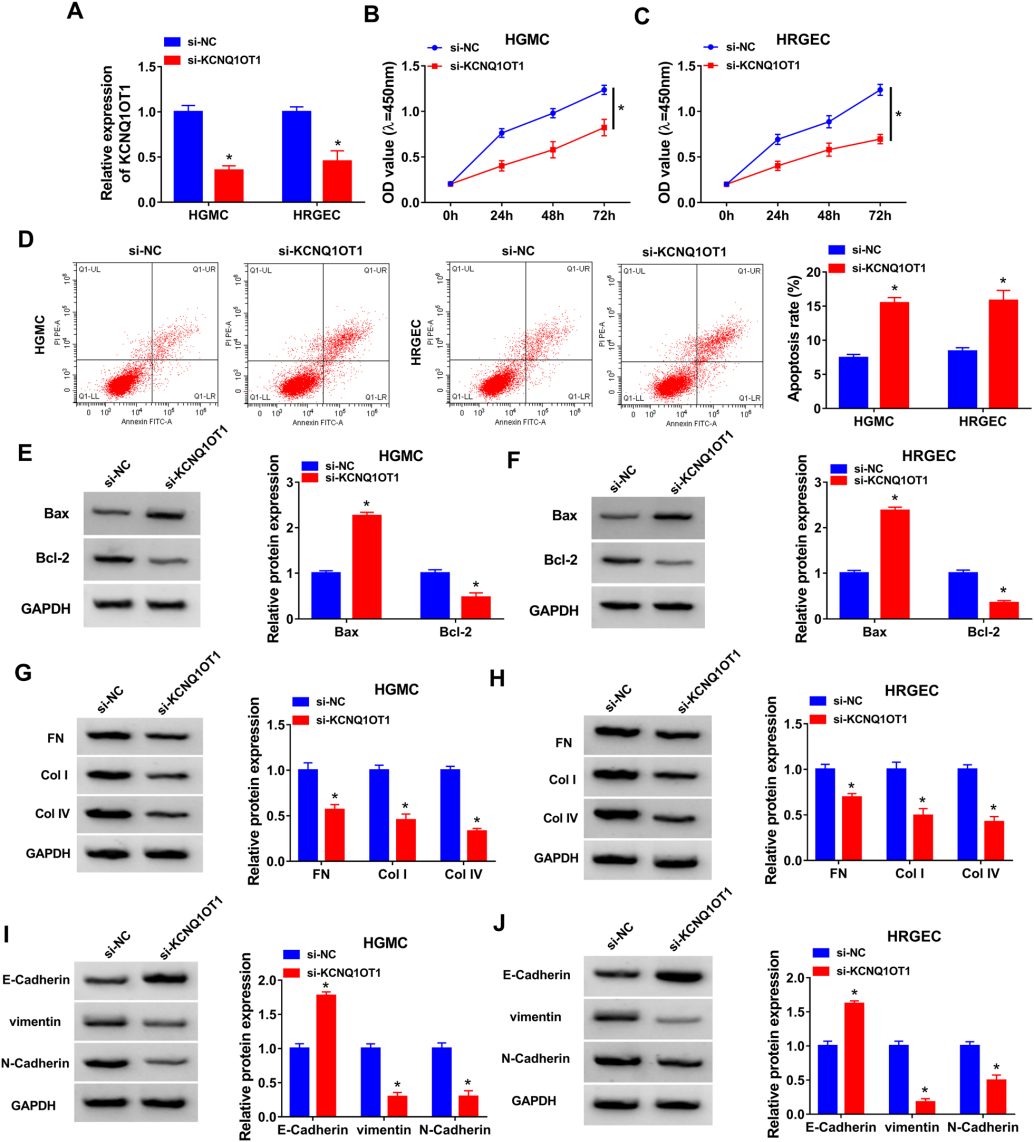
Knockdown of KCNQ1OT1 suppressed proliferation and fibrosis and induced apoptosis in HG-treated HGMC and HRGEC. HGMC and HRGEC were transfected with si-NC or si-KCNQ1OT1 and then treated with HG. **A** The expression of KCNQ1OT1 was examined by qRT-PCR. **B** and **C** Cell proliferation was assessed by CCK-8 assay. **D** Cell apoptosis rate was analyzed through the flow cytometry analysis. **E** and **F** Western blot assay was performed to detect the protein expression of Bax and Bcl-2. **G**–**J** The protein levels of FN, Col-I, Col-IV, E-Cadherin, vimentin, and N-Cadherin were measured by western blot assay. *P < 0.05

To study the effect of KCNQ1OT1 on proliferation, apoptosis and fibrosis in HG-induced HGMC and HRGEC, we transfected either si-KCNQ1OT1 or si-NC into cells then stimulated them with HG. The results of qRT-PCR showed that transfection of si-KCNQ1OT1 significantly reduced KCNQ1OT1 expression compared to si-NC group, suggesting a high transfection efficiency (Fig. 2A). CCK-8 assay showed that knockdown of KCNQ1OT1 reduced cell viability in HG-stimulated HGMC and HRGEC (Fig. 2B and C). Flow cytometry indicated that cell apoptosis was increased by transfection of si-KCNQ1OT1 in HG-treated HGMC and HRGEC (Fig. 2D). Meanwhile, KCNQ1OT1 deficiency increased the protein expression of Bax (pro-apoptotic molecule) and Cleaved-caspase 3 (a key executor in apoptotic process) and decreased the protein level of Bcl-2 (anti-apoptotic molecule) in HG-treated HGMC and HRGEC (Fig. 2E and F, Additional file 1: Figure S1A and B). Next, the protein levels of ECM (FN, Col-I and Col-IV) and EMT (E-Cadherin, vimentin and N-Cadherin) markers were determined using western blotting assay. The data indicated that the protein levels of FN, Col-I and Col-IV were decreased by knockdown of KCNQ1OT1 (Fig. 2G and H), suggesting that KCNQ1OT1 knockdown inhibited ECM accumulation. Moreover, KCNQ1OT1 downregulation promoted the protein expression of E-Cadherin (an epithelial marker) and inhibited the protein expression of vimentin and N-Cadherin (mesenchymal markers) (Fig. 2I and J), indicating inhibition of EMT progression. EMT can stimulate the excessive accumulation of ECM, which results in renal fibrosis [21]. Overall, KCNQ1OT1 knockdown might inhibit DN development.

### KCNQ1OT1 directly targeted miR-93-5p and negatively regulated miR-93-5p expression

**Fig. 3:**
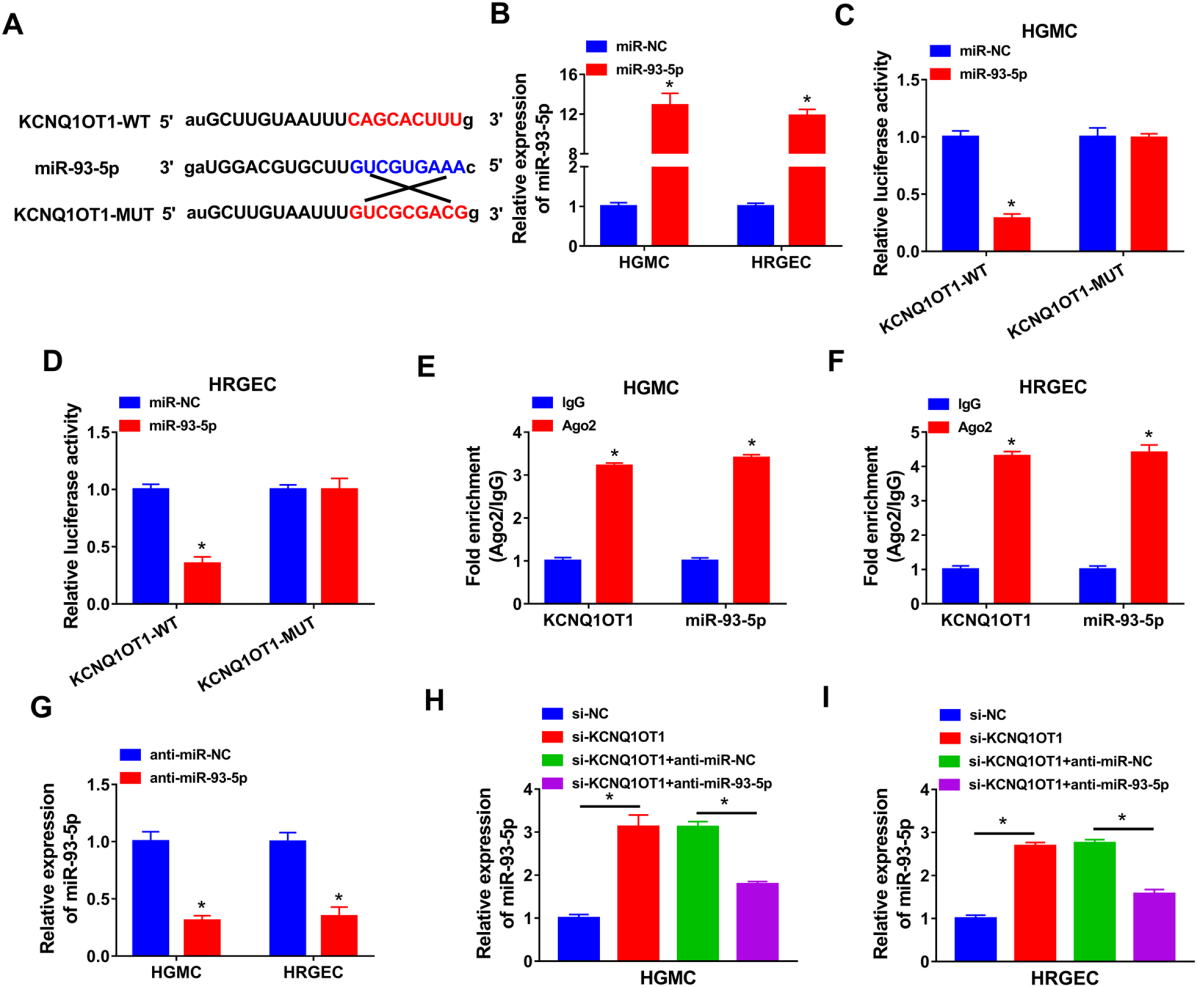
MiR-93-5p was a target of KCNQ1OT1. **A** The complementary binding sites between KCNQ1OT1 and miR-93-5p were predicted by starBase. **B** The expression of miR-93-5p was detected by qRT-PCR in HGMC and HRGEC transfected with miR-93-5p or miR-NC. **C** and **D** Dual-luciferase reporter assay was conducted to measure the luciferase activity in HGMC and HRGEC co-transfected with KCNQ1OT1-WT or KCNQ1OT1-MUT and miR-NC or miR-93-5p. **E** and **F** RIP assay was performed in HGMC and HRGEC and enrichment of KCNQ1OT1 and miR-93-5p was detected by qRT-PCR. **G** The expression of miR-93-5p was measured using qRT-PCR in HGMC and HRGEC transfected with anti-miR-NC or anti-miR-93-5p. **H** and **I** The expression of miR-93-5p was determined by qRT-PCR in HGMC and HRGEC transfected with si-NC, si-KCNQ1OT1, si-KCNQ1OT1 + anti-miR-NC, or si-KCNQ1OT1 + anti-miR-93-5p. *P < 0.05

To explore the relationship between KCNQ1OT1 and miR-93-5p, bioinformatics analysis (starBase) was performed. As presented in Fig. 3A, KCNQ1OT1 had binding sites with miR-93-5p. Overexpression efficiency of miR-93-5p was confirmed by qRT-PCR. Transfection of miR-93-5p markedly increased the expression of miR-93-5p in HGMC and HRGEC (Fig. 3B). To identify whether miR-93-5p could directly bind to KCNQ1OT1, dual-luciferase reporter assay and RIP assay were performed. The results showed that the luciferase activity of KCNQ1OT1-WT was obviously decreased in HGMC and HRGEC transfected with miR-93-5p, whereas the luciferase activity of KCNQ1OT1-MUT was unchanged after overexpression of miR-93-5p (Fig. 3C and D). Moreover, RIP assay exhibited higher enrichment levels of KCNQ1OT1 and miR-93-5p in Ago2 RIP group than those in IgG RIP group (Fig. 3E and F). Inhibition efficiency of miR-93-5p was validated by qRT-PCR in HGMC and HRGEC transfected with anti-miR-93-5p (Fig. 3G). Next, we explored the effect of KCNQ1OT1 on miR-93-5p expression. We found that the expression of miR-93-5p was increased by knockdown of KCNQ1OT1 in HGMC and HRGEC (Fig. 3H and I). These data demonstrated that KCNQ1OT1 directly interacted with miR-93-5p.

### KCNQ1OT1 exerted its biological function by targeting miR-93-5p in HG-induced HGMC and HRGEC

**Fig. 4:**
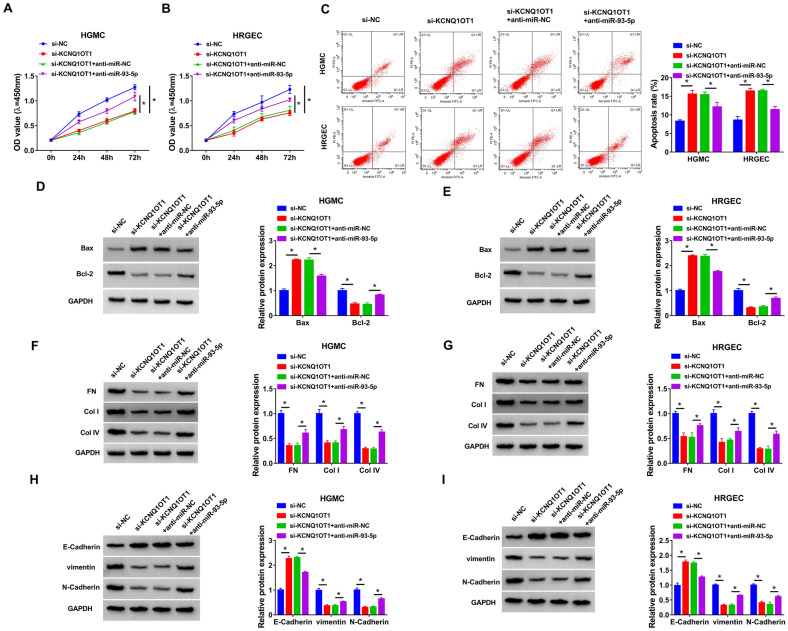
KCNQ1OT1 affected proliferation, apoptosis and fibrosis by sponging miR-93-5p HG-induced HGMC and HRGEC. HGMC and HRGEC were transfected with si-NC, si-KCNQ1OT1, si-KCNQ1OT1 + anti-miR-NC, or si-KCNQ1OT1 + anti-miR-93-5p and then treated with HG. **A** and **B** Cell proliferation was measured using CCK-8 assay. **C** Flow cytometry analysis was employed to determine apoptosis rate. **D** and **E** The protein levels of Bax and Bcl-2 were measured by western blot. **F**–**I** Western blot assay was performed to analyze the protein levels of FN, Col-I, Col-IV, E-Cadherin, vimentin, and N-Cadherin. *P < 0.05

To investigate whether the regulatory effect of KCNQ1OT1 was associated with miR-93-5p, HGMC and HRGEC were transfected with si-NC, si-KCNQ1OT1, si-KCNQ1OT1 + anti-miR-NC, or si-KCNQ1OT1 + anti-miR-93-5p before treatment of HG. CCK-8 assay indicated that miR-93-5p inhibition reversed the inhibitory effect of KCNQ1OT1 knockdown on cell proliferation in HG-treated HGMC and HRGEC (Fig. 4A and B). Moreover, miR-93-5p downregulation weakened si-KCNQ1OT1-induced apoptosis in HG-treated HGMC and HRGEC (Fig. 4C). In addition, the impact of KCNQ1OT1 silence on increasing Bax and Cleaved-caspase 3 protein expression and decreeing Bcl-2 protein expression was neutralized by downregulating miR-93-5p (Fig. 4D and E, Additional file 1: Figure S1C and D). Furthermore, downregulation of miR-93-5p abated the suppressive effect of KCNQ1OT1 knockdown on ECM accumulation by increasing FN, Col-I and Col-IV expression (Fig. 4F and G). Meanwhile, the effect of KCNQ1OT1 silence on promotion of E-Cadherin and reduction of vimentin and N-Cadherin expression was reversed by inhibition of miR-93-5p (Fig. 4H and I). Collectively, KCNQ1OT1 regulated proliferation, apoptosis and fibrosis in HG-induced HGMC and HRGEC by sponging miR-93-5p.

### ROCK2 was a direct target of miR-93-5p

**Fig. 5:**
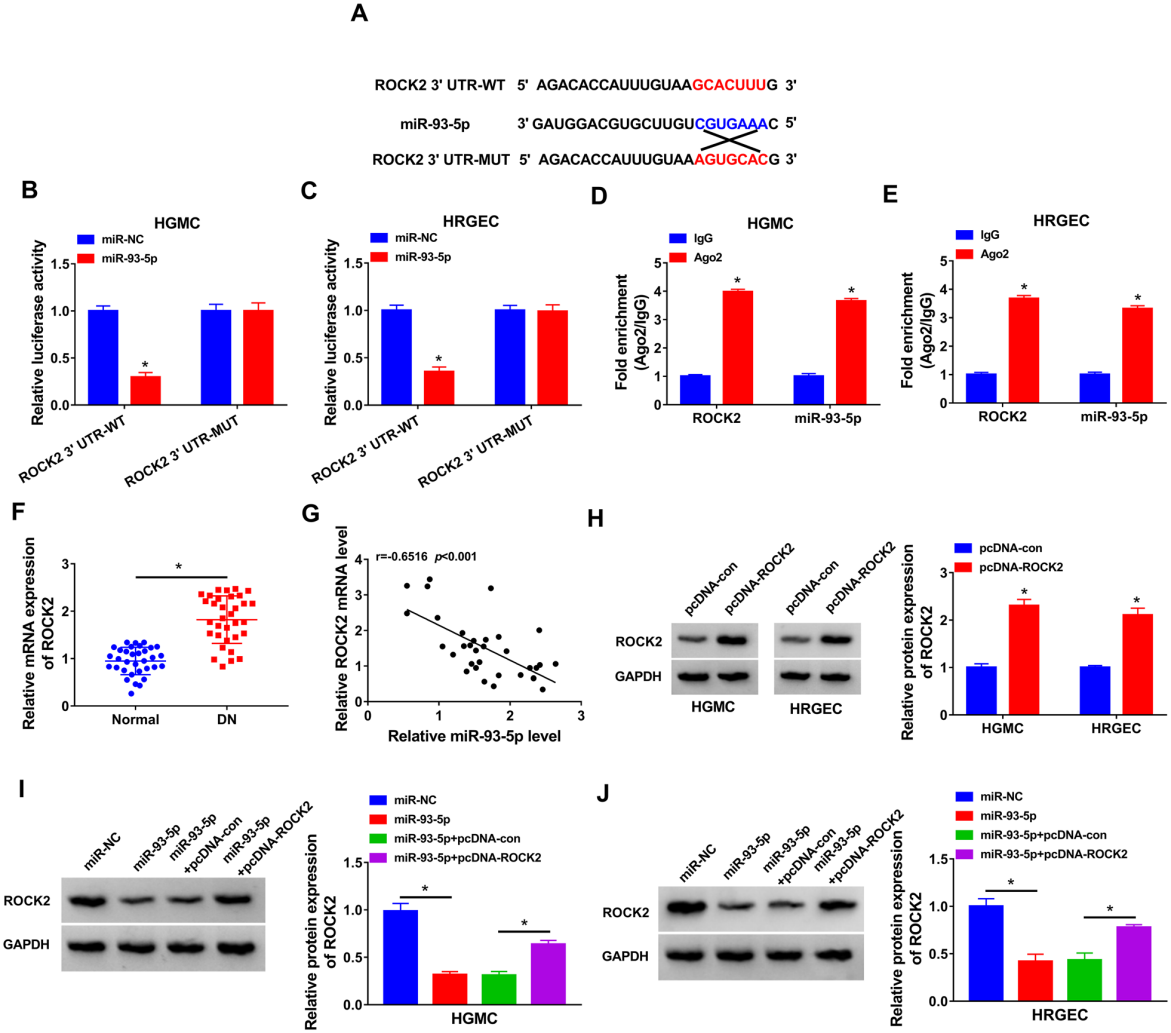
ROCK2 could directly bind to miR-93-5p. **A** TargetScan predicted the binding sites of ROCK2 and miR-93-5p. **B**–**E** Dual-luciferase reporter and RIP assays were performed in HGMC and HRGEC to validate the association between ROCK2 and miR-93-5p. **F** ROCK2 mRNA expression in the serum samples of DN patients was examine by qRT-PCR. **G** The correlation between ROCK2 mRNA expression and miR-93-5p expression was analyzed in serum samples of DN patients. **H** ROCK2 protein expression was detected by western blot assay in HGMC and HRGEC transfected with pcDNA-con or pcDNA-ROCK2. **I** and **J** The protein expression of ROCK2 was tested by western blot assay in HGMC and HRGEC transfected with miR-NC, miR-93-5p, miR-93-5p + pcDNA-con, or miR-93-5p + pcDNA-ROCK2. *P < 0.05

To further explore the mechanism, the targets of miR-93-5p were predicted by TargetScan. As presented in Fig. 5A, there were several binding sites between ROCK2 and miR-93-5p. The results of dual-luciferase reporter assay exhibited that miR-93-5p upregulation remarkably inhibited the luciferase activity of ROCK2 3′UTR-WT, but did not affect the luciferase activity of ROCK2 3′UTR-MUT (Fig. 5B and B). The data from RIP assay presented that both ROCK2 and miR-93-5p were greatly enriched in the RIP group with anti-Ago2 relative to the RIP group with anti-IgG (Fig. 5D and E). Moreover, ROCK2 mRNA expression was increased in the serum of DN patients (Fig. 5F). An inverse correlation between ROCK2 mRNA expression and miR-93-5p expression was observed in the serum samples of DN patients (Fig. 5G). Overexpression efficiency of ROCK2 was confirmed by western blot in HGMC and HRGEC transfected with pcDNA-con or pcDNA-ROCK2 (Fig. 5H). Additionally, overexpression of miR-93-5p reduced the protein expression of ROCK2 in HGMC and HRGEC, which was restored by upregulation of ROCK2 (Fig. 5I and J). All the data suggested that miR-93-5p directly targeted ROCK2.

### Overexpression of miR-93-5p suppressed proliferation and fibrosis and accelerated apoptosis in HG-induced HGMC and HRGEC

**Fig. 6:**
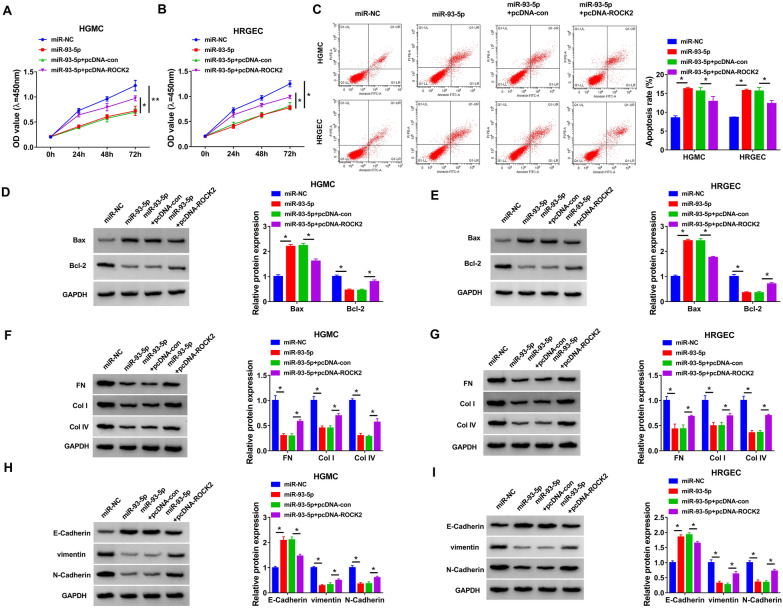
MiR-93-5p regulated proliferation, apoptosis and fibrosis by targeting ROCK2 in HG-stimulated HGMC and HRGEC. HGMC and HRGEC were transfected with miR-NC, miR-93-5p, miR-93-5p + pcDNA-con, or miR-93-5p + pcDNA-ROCK2, followed by treatment with HG. **A** and **B** Cell proliferation was assessed using CCK-8 assay. **C** Cell apoptosis was analyzed via flow cytometry analysis. **D** and **E** Western blot assay was conducted to examine the protein expression of Bax and Bcl-2. **F**–**I** FN, Col-I, Col-IV, E-Cadherin, vimentin, and N-Cadherin protein levels were measured by western blot assay. *P < 0.05

To evaluate the role of miR-93-5p and explored whether ROCK2 was involved in the function of miR-93-5p, HGMC and HRGEC were transfected with miR-NC, miR-93-5p, miR-93-5p + pcDNA-con, or miR-93-5p + pcDNA-ROCK2 and then treated with HG. The results showed that miR-93-5p overexpression inhibited cell viability and promoted apoptosis, which could be reversed by upregulation of ROCK2 (Fig. 6A–C). Also, the enhancement of Bax and Cleaved-caspase 3 protein expression and reduction of Bcl-2 protein expression caused by miR-93-5p could be counteracted by elevating ROCK2 (Fig. 6D and E, Additional file 1: Figure S1E and F). Moreover, enforced expression of miR-93-5p inhibited ECM accumulation by decreasing FN, Col-I and Col-IV expression, which was restored by overexpression of ROCK2 (Fig. 6F and G). In addition, miR-93-5p upregulation suppressed EMT progression by increasing E-Cadherin expression and decreasing vimentin and N-Cadherin expression, while this effect was abated by enhancement of ROCK2 (Fig. 6H and I). Taken together, miR-93-5p regulated proliferation, apoptosis and fibrosis in HG-induced HGMC and HRGEC by targeting ROCK2.

### KCNQ1OT1 regulated the expression of ROCK2 by sponging miR-93-5p

**Fig. 7:**
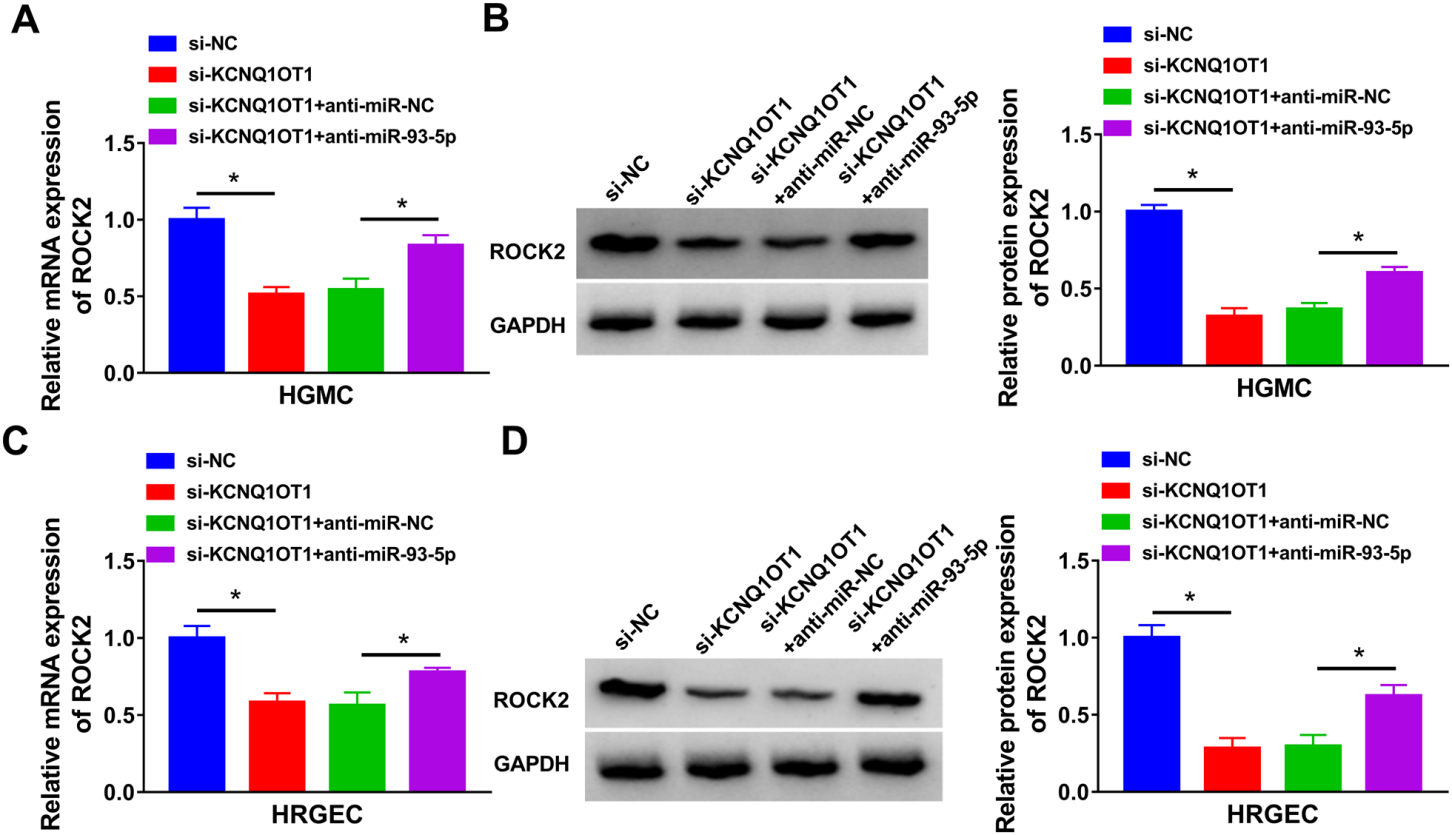
KCNQ1OT1 regulated ROCK2 expression by acting as a sponge of miR–93-5p. **A**–**D** The mRNA and protein expression of ROCK2 were determined by qRT-PCR and western blot in HGMC and HRGEC transfected with si-NC, si-KCNQ1OT1, si-KCNQ1OT1 + anti-miR-NC, or si-KCNQ1OT1 + anti-miR-93-5p. *P < 0.05

To explore whether KCNQ1OT1 acted as a sponge of miR-93-5p to regulate ROCK2 expression, HGMC and HRGEC were transfected with si-NC, si-KCNQ1OT1, si-KCNQ1OT1 + anti-miR-NC, or si-KCNQ1OT1 + anti-miR-93-5p. As displayed in Fig. 7A–D, KCNQ1OT1 silence reduced the mRNA and protein expression of ROCK2, which was restored by inhibiting miR-93-5p. These results suggested that KCNQ1OT1 positively regulated ROCK2 expression by targeting miR-93-5p.

## Discussion

DN is a primary microvascular complication of diabetes [22]. Many reports have shown that lncRNAs are involved in the initiation and development of DN and can be used as promising targets for DN treatment [23]. In the current study, we used the HG-stimulated HGMC and HRGEC to mimic DN and explore the potential role of lncRNA KCNQ1OT1 in DN.

KCNQ1OT1 has been suggested to be related to many diseases [24, 25]. Additionally, KCNQ1OT1 was reported to be upregulated in diabetic and KCNQ1OT1 knockdown inhibited pyroptosis in diabetic cardiomyopathy [26]. More importantly, KCNQ1OT1 was reported to be overexpressed in DN, and KCNQ1OT1 downregulation limited proliferation and fibrosis and accelerated apoptosis in DN cell models [14], indicating that KCNQ1OT1 might be related to the promotion of DN development. Nevertheless, the underlying mechanism of KCNQ1OT1 in pathogenesis of DN remains to be further explored. Here, we also found that KCNQ1OT1 was overexposed in serum samples of DN patients and DN cell model, which was consistent with previous study. Kidney fibrosis is considered to be the main pathologic change in DN, which eventually results in ESRD [27]. EMT process and ECM synthesis are the main features of kidney fibrosis [28–30]. To explore the biological role of KCNQ1OT1 in DN, loss-of-function experiments were performed. KCNQ1OT1 silence reduced cell proliferation, ECM accumulation and EMT and induced apoptosis in HG-induced HGMC and HRGEC, indicating that inhibition of KCNQ1OT1 might an effective strategy for the treatment of DN.

miR-93-5p is a well-explored miRNA, which has been disclosed to exert a critical role in cancers and diseases [17, 31–33]. Several research has disclosed that miR-93-5p exerted a protection effect in sepsis-induced acute kidney injury [34–36]. Wang et. al indicated that miR-93-5p constrained high glucose-induced malignant proliferation, fibrosis and inflammation in human glomerular mesangial cells [36]. Besides, miR-93-5p was disclosed to be regulated by XIST, thereby protecting against renal interstitial fibrosis in diabetic nephropathy [19]. In addition, miR-93-5p (miR-93) mediated Msk2-mediated high glucose-induced chromatin remodelling in the diabetic nephropathy, and overexpression of miR-93-5p constrained TGF-β1-induced EMT and renal fibrogenesis in DN [37, 38]. In consistent with previous research, miR-93-5p was lower expressed in serum of DN patients and DN cell models, and its expression was inversely correlated with KCNQ1OT1 level in serum of DN patients, implying that miR-93-5p and KCNQ1OT1 played different roles in DN. It is well known that that lncRNAs serve as ceRNAs to bind to miRNAs, thus reducing the miRNA-induced repression of their target mRNAs [39]. Based on ceRNA theory, we predicted the potential interaction between miR-93-5p and KCNQ1OT1 in DN by using starBase. As expected, miR-93-5p contained the complementary binding sites of KCNQ1OT1. Next, dual-luciferase reporter and RIP assays were conducted to verify this prediction. To investigate whether the functions of KCNQ1OT1 were mediated by miR-93-5p, rescue assays were carried out in DN cell models. We uncovered that miR-93-5p inhibition overturned the impact of KCNQ1OT1 deficiency on cell proliferation, apoptosis, ECM accumulation, and EMT in DN cell models, indicating that KCNQ1OT1 regulated DN progression through sponging miR-93-5p. Altogether, miR-93-5p exerted an inhibitory role in the development of DN.

**Fig. 8:**
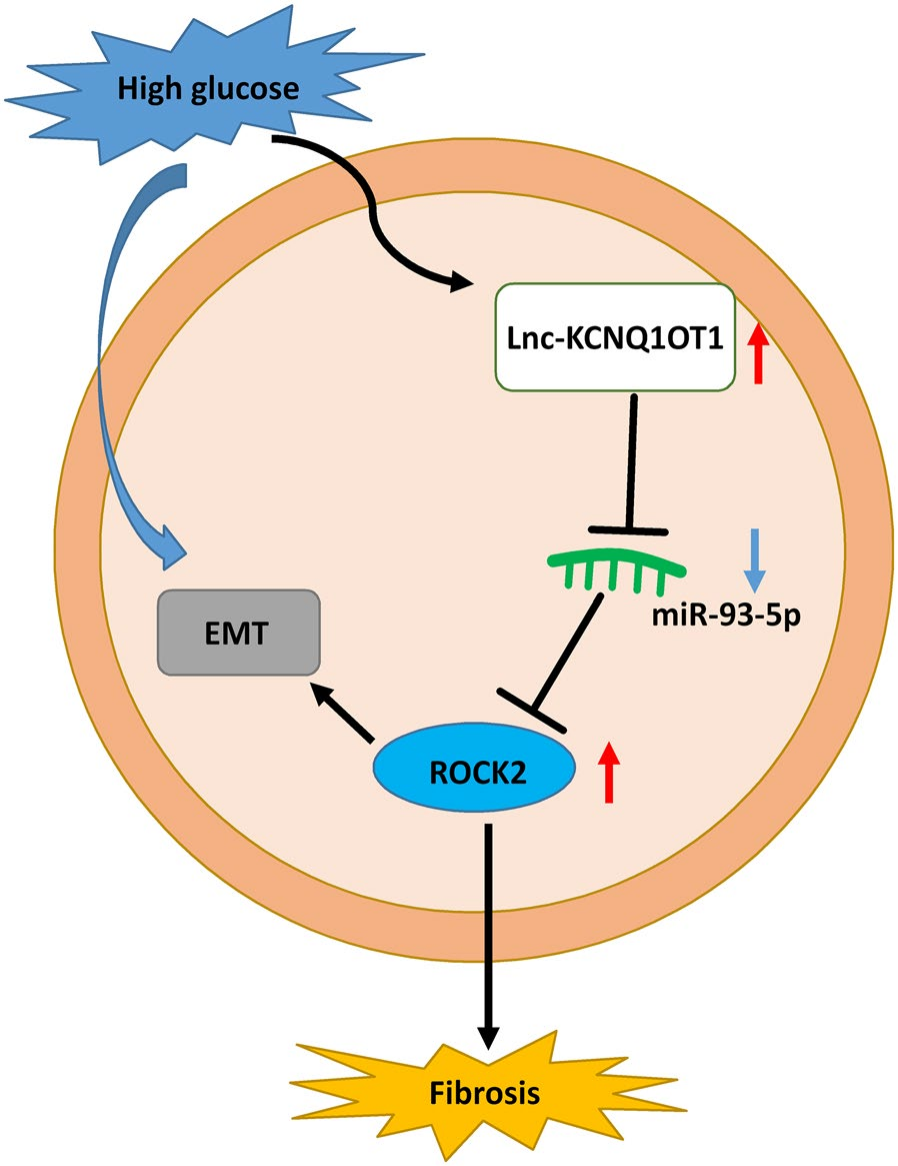
Schematic diagram illustrated the regulatory network of KCNQ1OT1/miR-93-5p/ROCK2 axis in HG-induced HGMC and HRGEC

To further study the regulatory mechanism of miR-93-5p in DN, bioinformatics tool was applied to predict the possible targets of miR-93-5p. ROCK2 was verified as a target for miR-93-5p. The biological function of ROCK2 related to DN was previously reported in some reports. For instance, melatonin reduced ROCK2 expression and activity in TGF-β2-stimulated glomerular endothelial cells to inhibit endothelial-to mesenchymal transition in DN [40]. Notably, ROCK2 overexpression reversed the repressive impact of miR-455-3p upregulation on proliferation, EMT and ECM accumulation in HG-induced cells [20]. These findings indicated that ROCK2 might promote DN development. In our paper, ROCK2 expression was enhanced in serum of DN patients and DN cell models, implying that ROCK2 might have the similar role with KCNQ1OT1 in DN. Rescue experiments indicated that ROCK2 upregulation abolished the effects of miR-93-5p on decrease of cell proliferation and fibrosis and increase of apoptosis in DN cell models, indicating that miR-93-5p might DN development by targeting ROCK2. Moreover, we observed that KCNQ1OT1 downregulation decreased ROCK2 expression via sponging miR-93-5p, indicating that KCNQ1OT1 served as a sponge for miR-93-5p to affect ROCK2 expression. Collectively, KCNQ1OT1 might promote DN development by regulating miR-93-5p/ROCK2 axis (Fig. 8).

In conclusion, this study demonstrated that KCNQ1OT1 silence suppressed proliferation and fibrosis and accelerated apoptosis in HG-stimulated HGMC and HRGEC by regulating miR-93-5p/ROCK2 axis. Our research provided novel insights into the mechanism of KCNQ1OT1 in HG-treated HGMC and HRGEC, which might broaden the understanding of the pathogenesis of DN. Nevertheless, future studies not only need to further clarify the role of KCNQ1OT1 in vitro cellular models, but also need to evaluate whether KCNQ1OT1 is also suitable to physiological functions in vivo*.* Moreover, the circRNAs/ miRNAs/mRNAs regulatory networks are very complicated, thus, the detailed physiological mechanisms for the effects of KCNQ1OT1 in DN need further exploration.

## Supplementary Information

**Additional file 1: Figure S1.** KCNQ1OT1 knockdown or miR-93-5p overexpression increased the protein expression of Cleaved-caspase 3 in HG-induced HGMC and HRGEC. (A and B) Western blot assay was carried out to detect the protein expression of Cleaved-caspase 3 in HG-induced HGMC and HRGEC transfected with si-NC or si-KCNQ1OT1. (C and D) The protein expression of Cleaved-caspase 3 was measured by western blot assay in HGMC and HRGEC transfected with si-NC, si-KCNQ1OT1, si-KCNQ1OT1 + anti-miR-NC, or si-KCNQ1OT1 + anti-miR-93-5p under HG conditions. (E and F) Cleaved-caspase 3 protein expression was examined using western blot assay in HGMC and HRGEC transfected with miR-NC, miR-93-5p, miR-93-5p + pcDNA-con, or miR-93-5p + pcDNA-ROCK2, and then treated with HG. *P < 0.05.

## Data Availability

Not applicable.
