# Supplementary figures and images for: LncRNA KCNQ1OT1 promotes the development of diabetic nephropathy by regulating miR-93-5p/ROCK2 axis
Source: Diabetol Metab Syndr. 2021 Oct 15;13:108. doi: 10.1186/s13098-021-00726-4 (PMC8518197; doi:10.1186/s13098-021-00726-4)

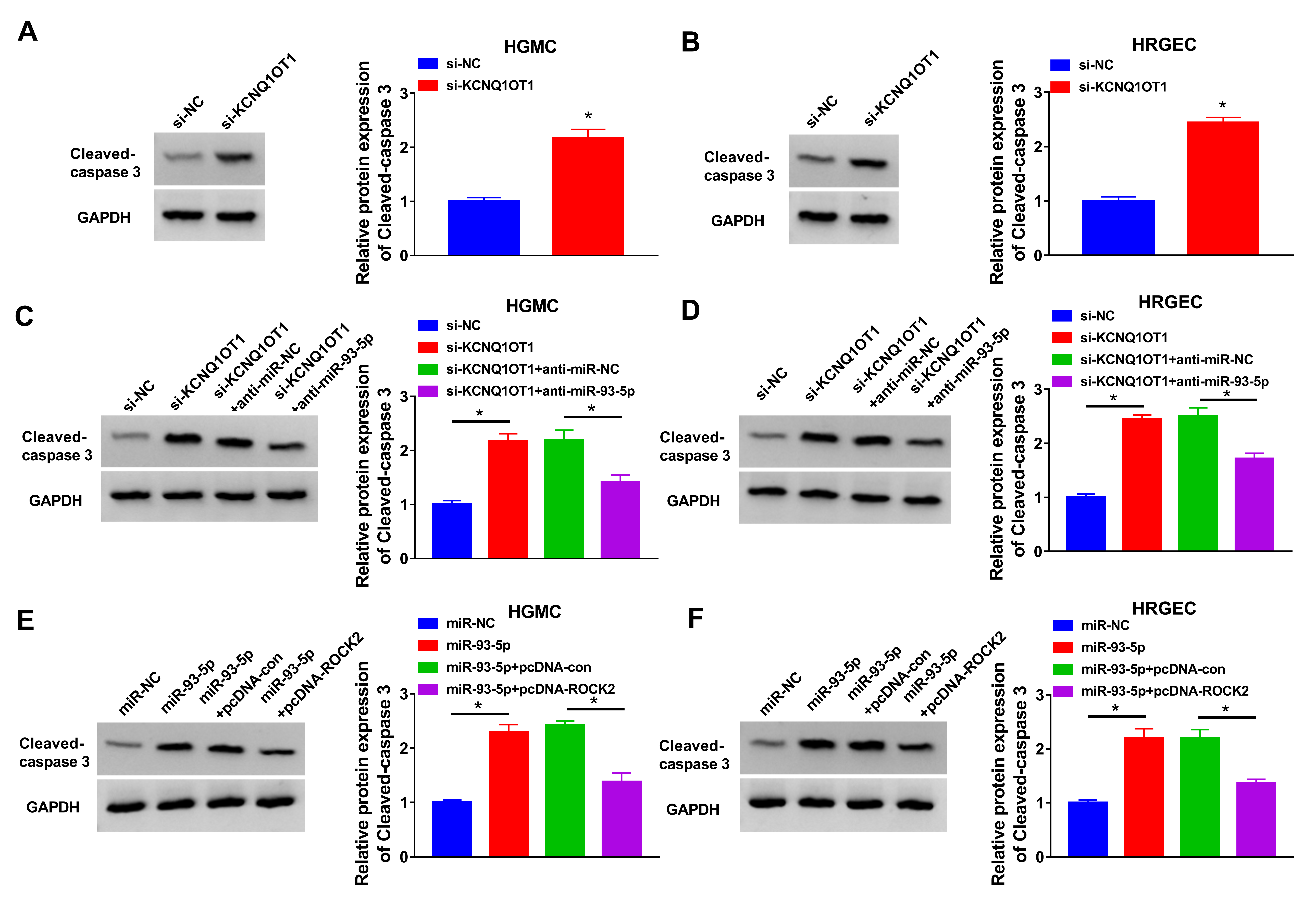

Supplement: Supplementary file 1 — Additional file 1: Figure S1. KCNQ1OT1 knockdown or miR-93-5p overexpression increased the protein expression of Cleaved-caspase 3 in HG-induced HGMC and HRGEC. (A and B) Western blot assay was carried out to detect the protein expression of Cleaved-caspase 3 in HG-induced HGMC and HRGEC transfected with si-NC or si-KCNQ1OT1. (C and D) The protein expression of Cleaved-caspase 3 was measured by western blot assay in HGMC and HRGEC transfected with si-NC, si-KCNQ1OT1, si-KCNQ1OT1 + anti-miR-NC, or si-KCNQ1OT1 + anti-miR-93-5p under HG conditions. (E and F) Cleaved-caspase 3 protein expression was examined using western blot assay in HGMC and HRGEC transfected with miR-NC, miR-93-5p, miR-93-5p + pcDNA-con, or miR-93-5p + pcDNA-ROCK2, and then treated with HG. *P < 0.05. [file 13098_2021_726_MOESM1_ESM.tif]
